# Mudd’s disease (MAT I/III deficiency): a survey of data for *MAT1A* homozygotes and compound heterozygotes

**DOI:** 10.1186/s13023-015-0321-y

**Published:** 2015-08-20

**Authors:** Yin-Hsiu Chien, Jose E. Abdenur, Federico Baronio, Allison Anne Bannick, Fernando Corrales, Maria Couce, Markus G. Donner, Can Ficicioglu, Cynthia Freehauf, Deborah Frithiof, Garrett Gotway, Koichi Hirabayashi, Floris Hofstede, George Hoganson, Wuh-Liang Hwu, Philip James, Sook Kim, Stanley H. Korman, Robin Lachmann, Harvey Levy, Martin Lindner, Lilia Lykopoulou, Ertan Mayatepek, Ania Muntau, Yoshiyuki Okano, Kimiyo Raymond, Estela Rubio-Gozalbo, Sabine Scholl-Bürgi, Andreas Schulze, Rani Singh, Sally Stabler, Mary Stuy, Janet Thomas, Conrad Wagner, William G. Wilson, Saskia Wortmann, Shigenori Yamamoto, Maryland Pao, Henk J. Blom

**Affiliations:** Department of Medical Genetics and Pediatrics, National Taiwan University Hospital, Children’s Hospital Building, Taipei, Taiwan; Division of Metabolic Disorders, CHOC Children’s, Orange, CA USA; Newborn Screening and Inborn Errors of Metabolism Regional Centre, Pediatric Endocrinology Program, Pediatric Unit, S.Orsola-Malpighi Hospital, University of Bologna, Bologna, Italy; Children’s Hospital of Michigan Metabolic Clinic, Detroit Medical Center, Detroit, MI USA; Department of Hepatology, Proteomics laboratory, Center for Applied Medical Research (CIMA), University of Navarra, IdiSNA, Pamplona, Spain; Head of Metabolic Unit, Department Pediatrics, Hospital Clínico Universitario de Santiago, Santiago de Compostela, Spain; Department of Gastroenterology, Hepatology and Infectious Diseases, Heinrich Heine University Düsseldorf, Düsseldorf, Germany; The Children’s Hospital of Philadelphia, Division of Metabolism, Perelman School of Medicine at the University of Pennsylvania, Philadelphia, PA USA; Department of Pediatrics, University of Colorado, Aurora, CO USA; Department of Clinical Sciences, Pediatrics Umeå University, SE 901 85 Umeå, Sweden; Department of Pediatrics, Division of Genetics and Metabolism; Department of Internal Medicine, Division of Clinical Genetics; and McDermott Center for Human Growth and Development, University of Texas Southwestern Medical Center, Dallas, TX USA; Department of Pediatrics, Shinshu University School of Medicine, 3-1-1, Asahi, Matsumoto, Japan; Division of Paediatrics, Department of Metabolic Diseases, Wilhelmina Children’s Hospital, University Medical Center Utrecht, Utrecht, The Netherlands; Department of Pediatrics, University of Illinois at Chicago, College of Medicine, Chicago, Il USA; Children’s Hospital Boston, Harvard Medical School, Boston, USA; KSZ Children’s Hospital/Korea Genetics Research Center, Jikjidaero, Heung Duck Gu, Cheng Ju City, Chung Buk Republic of Korea; Department of Genetics and Department of Metabolic Diseases, Hebrew University, Hadassah Medical Center, Jerusalem, Israel; Charles Dent Metabolic Unit, National Hospital for Neurology and Neurosurgery, London, UK; Department of General Pediatrics, Division of Pediatric Metabolic Medicine and Neuropediatrics, University Hospital Heidelberg, Heidelberg, Germany; Department of Neurology, University Children’s Hospital Frankfurt, Frankfurt, Germany; First Department of Pediatrics, University of Athens, Agia Sofia Children’s Hospital, Athens, Greece; Department of General Pediatrics, Neonatology and Pediatric Cardiology, University Children’s Hospital Duesseldorf, Duesseldorf, Germany; University Children’s Hospital, University Medical Center Hamburg Eppendorf, Hamburg, Germany; Department of Genetics, Hyogo College of Medicine, 1-1 Mukogawa-cho, Nishinomiya, Japan; Department of Medicine and Pathology, Biochemical Genetics Laboratory, Mayo Clinic College of Medicine, Rochester, MN USA; Department of Pediatrics and Laboratory Genetic Metabolic Diseases, Maastricht University Medical Center, Maastricht, Netherlands; Medical University of Innsbruck, Clinic for Pediatrics, Inherited Metabolic Disorders, Innsbruck, Austria; Genetics and Genome Biology, Peter Gilgan Center for Research and Learning The Hospital for Sick Children, Toronto, ON Canada; Department of Human Genetics and Pediatric, Emory University, Atlanta, GA USA; Department of Medicine, University of Colorado School of Medicine, Aurora, CO USA; Department of Medical and Molecular Genetics Indiana University School of Medicine, Indianapolis, IN USA; Department of Biochemistry, Vanderbilt University School of Medicine, Nashville, Tn USA; Division of Genetics, University of Virginia School of Medicine, Charlottesville, VA USA; Nijmegen Centre for Mitochondrial Disorders (NCMD), RadboudUMC, Amalia Children’s Hospital, Nijmegen, The Netherlands; Department of Pediatrics, National Shimoshizu Hospital, Chiba, Japan; Laboratory of Molecular Biology, National Institute of Mental Health, Bethesda, MD USA; Laboratory for Clinical Biochemistry and Metabolism, Center for Pediatrics and Adolescent Medicine University Hospital Freiburg, 79106 Freiburg, Germany

## Abstract

**Background:**

This paper summarizes the results of a group effort to bring together the worldwide available data on patients who are either homozygotes or compound heterozygotes for mutations in *MAT1*A. *MAT1A* encodes the subunit that forms two methionine adenosyltransferase isoenzymes, tetrameric MAT I and dimeric MAT III, that catalyze the conversion of methionine and ATP to *S*-adenosylmethionine (AdoMet). Subnormal MAT I/III activity leads to hypermethioninemia. Individuals, with hypermethioninemia due to one of the *MAT1A* mutations that in heterozygotes cause relatively mild and clinically benign hypermethioninemia are currently often being flagged in screening programs measuring methionine elevation to identify newborns with defective cystathionine β-synthase activity. Homozygotes or compound heterozygotes for *MAT1A* mutations are less frequent. Some but not all, such individuals have manifested demyelination or other CNS abnormalities.

**Purpose of the study:**

The goals of the present effort have been to determine the frequency of such abnormalities, to find how best to predict whether they will occur, and to evaluate the outcomes of the variety of treatment regimens that have been used. Data have been gathered for 64 patients, of whom 32 have some evidence of CNS abnormalities (based mainly on MRI findings), and 32 do not have such evidence.

**Results and Discussion:**

The results show that mean plasma methionine concentrations provide the best indication of the group into which a given patient will fall: those with means of 800 μM or higher usually have evidence of CNS abnormalities, whereas those with lower means usually do not. Data are reported for individual patients for *MAT1A* genotypes, plasma methionine, total homocysteine (tHcy), and AdoMet concentrations, liver function studies, results of 15 pregnancies, and the outcomes of dietary methionine restriction and/or AdoMet supplementation. Possible pathophysiological mechanisms that might contribute to CNS damage are discussed, and tentative suggestions are put forth as to optimal management.

## Background

More than sixty years ago Giulio Cantoni described an enzyme that utilized methionine and ATP to form a then novel product needed for transmethylation reactions [1], which he soon identified as *S*-adenosylmethionine (AdoMet) [2]. Since then, AdoMet has turned out to be among the most versatile compounds in all of biology. In humans AdoMet is used by as many as 200 or more methyltransferases [3]; after decarboxylation, as a source of aminopropyl groups in polyamine biosynthesis [4]; possibly as a source of other moieties [5]; and as a regulator of sulfur amino acid metabolism [6] and liver function [7]. Evidence has been presented that humans have a few enzymes that, as members of the group of more than 2800 proteins that comprise the biologically widespread radical SAM superfamily, use a 5′-deoxyadenosyl radical derived from AdoMet [8]. The structure of methionine adenosyltransferase (MAT)(E.C. 2.5.1.6), the enzyme that synthesizes AdoMet has been highly conserved during evolution: in a study of 292 *MAT* genes occurring in bacteria and eukaryota there was perfect conservation of active site residues, approximately 30 % of the encoded amino acids were identical in all species [9]. Humans possess two genes encoding isoforms of MAT: *MAT1A* encodes a catalytic subunit that forms the tetrameric and dimeric holoenzymes, MAT I and MAT III. *MAT2A* encodes the catalytic subunit of MAT II [10]. Sánchez-Pérez et al. found that each vertebrate included in their study had two *MAT* genes that encoded amino acid sequences with about 85 % identity, suggesting a gene duplication that occurred after the human lineage diverged from the sea squirts (Urochordata), but before divergence from the jawed vertebrates (Teleostomi) [9], perhaps 400–500 million years ago. MAT II is the major isozyme in non-hepatic tissues and in fetal liver, whereas MAT I and III are the major forms present in post-natal liver [10]. However, *MAT1A* expression has been reported in pancreas [11] and, more recently, in small amounts in most tissues, including brain [12]. After the introduction of screening newborns for methionine elevations to detect homocystinuria due to cystathionine β-synthase deficiency, infants were discovered in the United States [13], France [14], and Japan [15] with elevated blood methionine but without abnormal levels of homocystine, and MAT activities were shown to be low in extracts of their livers [13, 15–18], as well as in that of an adult patient found to be hypermethioninemic when investigated because of malodorous breath due to dimethylsulfide [19, 20]. The kinetic properties of the residual hepatic MAT activities of these patients [16, 19] as well as the finding that MAT activities in a variety of their non-hepatic tissues were normal indicted that the defective activity was that encoded by *MAT1A* [16, 17, 21]. Establishment of the amino acid sequence encoded by *MAT1A* [22–24] then opened the way to finding *MAT1A* mutations in most of the patients in question [25–27]. As screening of newborns for methionine elevations has expanded, *MAT1A* mutations are turning out to be the most common genetic cause of newborn hypermethioninemia [28–31]. Most such cases are heterozygous for R264H, a mutation in which the activity of wild-type subunits is suppressed by combination with mutant subunits [32, 33]. Several other such dominant mutations have now been identified [31, 34, 35]. Clinically, heterozygotes for such mutations have been unaffected. However, in addition, a number of patients with homozygous or compound heterozygous *MAT1A* mutations have been found through newborn screening [30, 31, 36–44], and although some of these patients have been clinically unaffected, some of them have had brain demyelination or other MRI abnormalities. Treatments used have included dietary methionine restriction or supplementation with AdoMet, but optimal management has not yet been clearly established. With the aim of providing information on the criteria for prognosis and optimal treatment of such patients we have recently made an effort to collect as much information as possible on the clinical status and outcomes and the metabolic details of patients for whom sequencing has shown either homozygous or compound heterozygous *MAT1A* mutations, or those with hypermethioninemia and deficient MAT activities. The findings are presented and discussed in this paper.

## Methods

A literature search was carried out to identify patients who had been found to be homozygotes or compound heterozygotes for *MAT1A* mutations or who, although not genotyped, had been shown to have deficient activities of hepatic MAT. Information on such patients was taken from relevant publications and analyzed together, whenever possible, with updated information submitted by coauthors based on their experiences in management of, or diagnostic studies of, the patients in question. In addition, physicians taking care of MAT I/III patients came known to the authors via support in the diagnostic process or counseling on potential treatment of these patients. Informed consent was obtained from the patients or their parents and local ethical rules were followed. Ethical approval was obtained from the Office of Human Subjects Research (OHSR) at the NIH, Bethesda, USA.

Patients were considered to have “CNS abnormality” if their brain MRI showed abnormalities or if presence of any neurological symptom was reported or both.

In Tables 1 and 2 the references are given of those patients that have been published before.

**Table 1:** Patients without evidence of CNS abnormalities

Patient	Sex	Age at last report years	MAT1A allele 1/allele 2	MAT activity % of WT	Cause ascer- tain Met	Age < 5 m Met range	Age ≥ 5 m Met mean ± SD	MRI (age)	Citation^†^
G1	F	40.4	c.966T>G (p.Ile322Met)	7.8*	NBS	1270	373 ± 133	Normal (22)	[17, 25, 46, 58]
			c.966T>G (p.Ile322Met)	21/21					
G2	M	38	c.914T>C (p.Leu305Pro)	10.2*	NBS	364–583	394 ± 118	---	[17, 25]
			c.966T>G (p.Ile322Met)	26/21					
G3	F	2	c.164C>A (p.Arg55Asp)	10.91*	NBS	389–1020	315	---	[17, 25]
			c.1070C>T (p.Pro357Leu)	26/31					
G4	F	31.4	c.1068G>A (p.Arg356Gln)	17.5*	NBS	42–408	146 ± 38	---	[17, 26]
			c.1132G>A (p.Gly378Ser)	11/0.2	402				
2	M	60.1	c.539insTG (p.Thr185X)	28*	Breath odor	---	805 ± 106	Normal (31)	[19, 20, 78]
			c.539insTG (p.Thr185X)						
3	F	21.5	c.539insTG (p.Thr185X)	NA	NBS	1870–2542	777 ± 169	Normal (6)	[26, 58]
			c.539insTG (p.Thr185X)		1870				
10^a^	M	25	c.595C>T (p.Arg199Cys)	11/11	NBS	496–670	520 ± 124	---	[26, 58]
			c.595C>T (p.Arg199Cys)		670				
11^a^	F	28.5	c.595C>T (p.Arg199Cys)	11/11	NBS	591–654	475 ± 118	---	[26, 58]
			c.595C>T (p.Arg199Cys)		591				
14	F	6.4	c.966T>G (p.Ile322Met)	11/11	NBS	440–467	225 ± 57	---	[27, 58]
			c.1031A>C (p.Glu344Ala)						
15^b^	F	2.2	c.1132G>A (p.Gly378Ser)	0.2/75	NBS	383–1089	519	---	[80]
			c.1161G>A (p.Trp387X)						
16^b^	F	4.7	c.1132G>A (p.Gly378Ser)	0.2/75	Family	---	782 ± 400	---	[80]
			c.1161G>A (p.Trp387X)						
17	F	21.4	c.791C>T (p.Arg264Cys)	0.3/31	NBS	648	322	---	[42]
			c.1070C>T (p.Pro357Leu)						
18	M	21.2	c.1070C>T (p.Pro357Leu)	31/31	NBS	505	325 ± 244	---	[42]
			c.1070C>T (p.Pro357Leu)						
19	F	16.5	c.1067G>C (p.Arg356Pro)	11/31	NBS	622	175	---	[34, 42]
			c.1070C>T (p.Pro357Leu)						
21	F	7.1	c.836G>T (p.Gly279Val)	NA/2	NBS	596	436 ± 1	---	[34]
			c.964A>G (p.Ile322Val)						
23	F	3.9	c.539insTG (p.Thr185X)	NA	NBS	216–1457	665 ± 160	---	[38]
			c.822G>C (p.Trp274Ser)						
24^c^	M	1.2	c.689T>G (p.Val230Gly)	NA	NBS	518–525	769 ± 112	---	
			c.689T>G (p.Val230Gly)						
25^c^	F	4.9	c.689T>G (p.Val230Gly)	NA	Family	–	838 ± 18	---	
			c.689T>G (p.Val230Gly)						
26	F	2.9	c.527T>A (p.Leu176Gln)	NA	NBS 57	57–200	76 ± 24	---	
			c.527T>A (p.Leu176Gln)						
28	F	20	c.65C>T (p.Ser22Leu)	46/46	NBS	---	975 ± 177	normal (20)	[34, 37, 44]
			c.65C>T (p.Ser22Leu)						
33	U	2	c.856G>A (p.Asp286Asn)	NA	NBS	106–1570			
			del at least exons 6-8						
40	F	0.4	c.1141G>A (p.Gly381Arg)	25/25	NBS	1207–2121	1673	---	[34]
			c.1141G>A (p.Gly381Arg)						
44	M	0.2	c.529C>T (p.Arg177Trp)	NA	NBS 97	97–641	---	---	
			c.529C>T (p.Arg177Trp)						
48	M	5.4	c.446T>A (p.Met64Lys)	NA	NBS 134	134–1567	---	Normal (yearly, final at 5) DQ 88	[39]
			c.589delC (p.Pro197Leufs*26)						
49	F	0.3	c.110T>C (p.Ile37Thr)	NA	NBS 407	403–408	---	---	[31]
			c.271G>A (p.Gly91Ser)						
52	M	0.54	c.823G>C (p.Gly275Arg)	NA	NBS	949–1074	---	normal (0.5)	
			c.823G>C (p.Gly275Arg)						
53	F	2.3	c.862A>G (p.Thr288Ala)	NA	NBS	121–484	495 ± 275		[30]
			c.862A>G (p.Thr288Ala)						
54	F	2.0	c.1064T>G (p.Leu355Arg)	NA	NBS 185	185–410	---	---	[30]
			c.1064T>G (p.Leu355Arg)						
57	U	3.5	c.169+1G>A	NA	NBS 860	860–1130	918 ± 84	---	
			c.169+1G>A						
58^d^	M	12.3	not sequenced	22.7*	NBS	268–1005	369 ± 237	---	[15]
					268				
59^d^	M	13.7	not sequenced	23.1*	Family	---	350 ± 50	---	[15]
64	M	4.5 m	c.596G>A (p.Arg199His)	NA	NBS	118–208	---	---	
			c.596G>A (p.Arg199His)						

^†^Patients are assigned the same identifiers as were used when they were first described in the following papers: Patients G1, G2, G3, G4 [17]; Patients 3, 10, 11 [58]; Patients 17, 18, 19; numbers 1, 2 and 5 [42]; Patient 28: patient 1 [44]; Patients 53, 54: patients 14 and 18 [30]

*Activity based on assay of hepatic extract; others based on activity of mutant recombinant forms expressed in *E coli* [34]

^a^Sibs are designated by the same superscript

^†^Cont. indicates that methionine restriction or AdoMet supplementation was continuing at last report

NBS: Newborn screening

NA: not available

Met: methionine concentration in umol/L

**Table 2:** Patients with evidence of CNS abnormalities

Patient	Sex	Age last report years	MAT1A allele 1/allele 2	MAT activity % of WT	Cause ascer- tain-ment	Age < 5 m Met range	Age ≥ 5 m Met mean ± SD	MRI (age done) Other	Citation^†^
1	M	24.2	c.113G>A (p.Ser38Asp)	8*	NBS	670–1237	1030	Abnormal (20.7)	[14, 16, 27, 58]
			c.255delCA (p.Tyr92X)	0/NA	670				
4	F	32	c.827insG (p.Lys351X)	NA	NBS	---	600–1400	Abnormal (11)	[26, 64]
			c.827insG (p.Lys351X)					Normal (12)	
5	F	26.1	c.791C>T (p.Arg264Cys)	0.3/23	NBS	201–1740	909 ± 272	Normal (13)	[27, 47, 58]
			c.1006G>A (p.Gly336Arg)		201			IQ 84 (14)	
								Learning disability (14)	
7	M	17.8	c.292G>A (splicing)	NA	NBS	1226–1664	1428 ± 320	Normal (0.75)	[27, 58]
			c.292G>A (splicing)		1226			Abnormal (4.2)	
								Abnormal (11)	
8	F	13.6	c.1043delTG (p.His350X)	NA	Dystonia	---	1157 ± 445	Abnormal (9)	[26, 58]
			c.1043delTG (p.His350X)						
9	M	8.7	c.595C>T (p.Arg199Cys)	11/NA	NBS	966–1467	879 ± 140	Normal (6)	[26, 58]
			c.539insTG (p.Thr185X)					IQ 65 (6)	
13	F	6.2	c.595C>T (p.Arg199Cys)	11/11	NBS	635–738	485 ± 49	Abnormal (8)	[26, 58]
			c.595C>T (p.Arg199Cys)					Slow at school (16)	
20	F	10.8	c.205G>A (p.Gly69Ser)	109/NA	NBS	---	1400	Abnormal (10)	[42]
			c.1188G>T (p.X396YfsX464)		1560				
22	F	7.5	c.874C>T (p.Arg292Cys)	14/NA	NBS	---	1005–1676	Abnormal (2.9)	[38, 81]
			c.1067G>T (p.Arg356Leu)		395			Normal (6.4)	
								IQ 60 (5.4)	
29	M	14.2	c.125T>C (p.Leu42Pro)	10/10	NBS	121–1541	1437 ± 498	Abnormal (13)	[34, 37, 44]
			c.125T>C (p.Leu42Pro)		121			IQ 73 (13)	
30^e^	M	7.0	c.274T>C (p.Tyr92His)	104/11	NBS	740	830 ± 368	IQ 121 (3)	[34, 36, 41]
			c.1067G>C (p.Arg356Pro)					Abnormal (3.8)	
								Better (5.5)	
								Normal (7)	
31^e^	M	3	c.274T>C (p.Tyr92His)	104/11	NBS	820–2250	900–1140	Abnormal (0.8)	[34, 41]
			c.1067G>C (p.Arg356Pro)					Abnormal (3)	
32	M	9.4	c.433G>A (p.Glu145Lys)	NA/14	NBS	1740–1870	740–1150	Abnormal (5)	[34, 43]
			c.874C>T (p.Arg292Cys)					Abnormal (9.4)	
								IQ 108 (9.4)	
34	M	0.63	c.1068G>A (p.Arg356Trp)	4/4	NA	1846–3500	---	Severe retarded	[34]
			c.1068G>A (p.Arg356Trp)						
35	F	2.8	c.292G>A (splicing)	NA/11	NBS	1544–1685	1159	Abnormal (1.2)	[26]
			c.595C>T (p.Arg199Cys)					Better (1.8)	
36^f^	F	1.3	c.539insTG (p.Thr185X)	NA/20	NBS†	1400	1549	Normal (2.3)	[34]
			c.890C>A (p.Ala297Asp)		1400			Delayed development (2.3)	
37^f^	F	1.3	c.539insTG (p.Thr185X)	NA/20	NBS† 1400	1400–1421	1614	Delayed development (2.3)	[34]
			c.890C>A (p.Ala297Asp)						
38^g^	F	13	c.896G>A (p.Arg299His)	13/13	Neuro-logical	--	1016 ± 549	Abnormal (12.8)	[34]
			c.896G>A (p.Arg299His)					Delayed development (3)	
39^g^	M	9.8	c.896G>A (p.Arg299His)	13/13	Family	---	1000 ± 192	Abnormal (5.2)	[34]
			c.896G>A (p.Arg299His)					Delayed in learning (10.5)	
41	F	7.6	p.MAT1Adel	NA	NBS	192–1608	---	Abnormal (0.2)	
			p.MAT1Adel		192			Normal (7.1)	
42	M	1.9	c.607delATC (p.Ile203del)	NA	NBS	148–1490	1144 ± 143	Abnormal (1.2)	
			c.607delATC (p.Ile203del)		148			Speech delay (1.5)	
43	M	4.2	c.934C>T (p.Arg312Trp)	NA	NBS	460–1437	1120	Abnormal (3.8)	
			c.934C>T (p.Arg312Trp)					IQ 78 (3.3)	
45	F	4.5	c.292G>C (p.Gly98Arg, splicing?)	NA	NBS	507–1012	901 ± 163	Abnormal (4)	
			c.292G>C (p.Gly98Arg, splicing?)					Better (4.5)	
46	F	34	c.274T>C (p.Tyr92His)	8.3*	NBS	402–1340	1326 ± 159	Abnormal (30)	[15]
			c.1067G>C (p.Arg356Pro)	104/11				IQ 99 (9)	
47	F	17	c.1033insG (p.Lys351X)	NA	Unknown	---	---	Abnormal (9)	
			c.1033insG (p.Lys351X)					Almost normal (17)	
50^h^	F	4.9	c.896G>A (p.Arg299His)	NA	Neuro-logical	---	878 ± 136	Abnormal (3.3)	[34]
			c.896G>A (p.Arg299His)						
51^h^	F	4.7	c.896G>A (p.Arg299His)	NA	Family	---	641 ± 74	Behavior deterioration (3.7)	[34]
			c.896G>A (p.Arg299His)						
56	F	39	c.896G>A (p.Arg299His)	NA	Hi met baby	---	1233 ± 513	Neurological abnormality	
			c.896G>A (p.Arg299His)						
60	F	6.1	c.895C>T (p.Arg299Cys)	20/20	NBS	800–1067	1013 ± 283	Abnormal (1.4)	[34, 40]
			c.895C>T (p.Arg299Cys)					Mild delay (5.6)	
61	F	1.0	c.688G>A (p.Val230Met)	NA /11	NBS	205–328	84 ± 34	Abnormal (4.5)	[82]
			c.1067G>C (p.Arg356Pro)					Neurologic normal (5.5)	
62	M	4.3	c.169G>A (p.Glu57Lys, splicing?)	NA	NBS	461	812 ± 309	Developmental delay (2.3)	
								MRI normal (2.3)	
								Development normal (4.3)	
63	M	2.9	c.169-2A>c.734_735delAG (p.Gln245Profs*20)G	NA/0.3	NBS	938–1271	905 ± 99	Abnormal (0.8)	
			c.791C>T (p.Arg264Cys)					Worsen (1.5)	

^†^Patients are assigned the same identifiers as were used when they were first described in the following papers: patient 1 [14, 16]; patients 5, 7, 8, 9 and 13 [58]; patient 20: 14 [42]; patient 22 [38]; patient 29: patient 2 [44]; patient 30 [36]; patient 31 [41]; patient 32 [43]; patient 46: case 2 [15], and patient 61 [82]

*Activity based on assay of hepatic extract; others based on activity of mutant recombinant forms expressed in *E coli* [34]

^a^ Sibs are designated by the same superscript

^†^Cont. indicates that methionine restriction or AdoMet supplementation was continuing at last report

NBS: Newborn screening

NA: not available

Met: methionine concentration in umol/L

## Results

### Summary tables

Currently data are available for 64 patients. Because a substantial portion of these patients have evidence of CNS abnormalities, we divided them between those without (Table 1) (*n* = 32) and with evidence of CNS abnormalities (Table 2) (*n* = 32). These Tables show many more important features of the patients, including their *MAT1A* genotypes, their age at last report, cause of ascertainment, plasma methionine concentrations and whether MRI’s, if performed, were normal or abnormal. Tables 3 and 4 present the brief clinical histories of the patients with possible CNS abnormalities, the interventions used (if any), and the MRI findings at specified ages.

**Table 3:** Outcomes for treated patients

Patient #	Diet	Time period		Met μM	MRI	Clinical
		Start years	End years	Mean ± SD (N)		
Patients without evidence of CNS problems who had periods of dietary methionine restriction						
3	Normal	Birth	4.5 m	2262 ± 350 (3)		
	Restricted	4.5 m	3.4 y	462 ± 70 (5)		
	Normal	3.4 y	21.5 y	777 ± 169 (8)	Normal (6 y)	Normal (21.5 y)
17	Normal	Birth	0.8 m	648 (1)		
	Restricted	0.8 m	21.4 y	282 ± 42 (3)		
	Normal	21.4 y	22 y	314 (1)	n.d.	Normal (21.4 y)
18	Normal	Birth	1 m	305 (1)		
	Restricted	1 m	14 y	135 ± 40 (3)		
	Normal	14 y	21.2 y	327 ± 241 (2)	n.d.	Normal (21.2 y)
19	Normal	Birth	1 m	622 (1)		
	Restricted	1 m	16.4 y	156 ± 36 (5)		
	Normal	16.4 y	16.5 y	175 (1)	n.d.	Normal (16.5 y)
21	Normal	Birth	0.4 m	596 (1)		
	Restricted	0.4 m	5.8 y	282 ± 95 (6)		
	Normal	5.8 y	7.1 y	436 ± 1 (2)	n.d.	Normal (7.1 y)
48	Normal	Birth	1 m	643 ± 646 (3)		
	Restricted	1 m	5.4 y	545 ± 118 (50)	n.d.	Normal (5.4 y)
53	Normal	Birth	4.3 m	303 ± 257 (2)		
	Normal	5.2 m	1.2 y	495 ± 275 (4)		
	Restricted	1.2 y	2.3 y	266 ± 96 (3)	n.d.	Normal (2.3 y)
58		Birth	1 m	783 ± 337 (3)		
	Restricted	1 m	6 m	134–268 (2)		
	Normal	6 m	6.5 m	536 (1)		
	Restricted	6.5 m	6 y	67–268 (2)		
	Normal	6 y	36 y	263 ± 83 (7)	n.d.	No problems (6 y)
Patients with evidence of CNS problems who had periods of dietary methionine restriction						
1	Normal	Birth	2 m	954 ± 401 (2)		
	Restricted	2 m	24 y	100–600 (2)	Abnormal (20.7 y)	Normal intelligence (24 y)
5	Normal	Birth	3 m	1345 ± 661 (5)		
	Restricted	6 m	2.5 y	899 ± 129 (4)		Considered slow
	Normal	2.5 y	26.1 y	909 ± 272 (3)	Normal (13 y)	Wechsler Intelligence Scale verbal 88, performance 84 (7.5 y)
9	Normal	Birth	1 m	1217 ± 354 (2)		
	Restricted	1 m	1.2 y	220 ± 176 (10)		
	Normal	1.2 y	8.7 y	879 ± 140 (5)	Normal (6 y)	IQ 65 (1^st^ percentile) (6 y)
13	Normal	Birth	1.3 m	697 ± 87 (2)		
	Restricted	1.7 m	10 m	193 ± 83 (4)		
	Normal	10 m	10 y	485 ± 49 (4)	Abnormal (8 y)	Slow at school (16 y)
20	Normal	Birth	1.5 m	1528 ± 85 (4)	Abnormal (0.1 y)	
	Restricted	1.5 m	6.6 y	684 ± 234 (14)	Normal (4.9 y)	Normal (5.4 y)
	Normal	6.6 y	10.5 y	1298 ± 112 (5)	Abnormal (10 y)	
	Restricted	10.5 y	10.8 y	1180 (1)		
30	Normal	Birth	0.6 m	740		
	Restricted + betaine	0.6 m	2.5 y	400–1580		
	Restricted + betaine + B6	2.5 y	3.9 y	1080–1370	Abnormal (3.8 y)	IQ 121 (3.5 y)
	Normal	3.9 y	9 y	1050	Improved (5 y) better (7 y)	
31	Normal	Birth	3 m			
	Restricted	3 m	1.8 y		Abnormal (0.8y)	Development normal
					Abnormal (3 y)	
32	Normal	Birth	NA	1805 ± 92 (2)		
	Restricted	NA	5.2 Y	740–1150	Abnormal (5 Y)	
	Normal	5.2 Y	9.4 Y	NA	Abnormal (9.4 y)	IQ 108 (9.4 y); neurologically normal
43	Normal	Birth	4 m	949 ± 691 (2)		
	Normal	4 m	6 m	1145		
	Restricted	9.6 m	3.2 y	928 ± 74 (8)		
	Normal	3.2 y	3.8 y	1120 (1)	Abnormal (3.8 y)	Performance IQ 78; reasoning 57 (3.3 y)
	Restricted	4 y	4.2 y	994 (1)		
46	Normal	Birth	1.5 m	1027 ± 542 (3)		
	Restricted	1.5 m	4 y	332 ± 341 (28)		
	Normal	4 y	34 y	1326 ± 159 (29)	Abnormal (30 y)	Physical and mental development normal (34 y)
61	Normal	Birth		735		Normal
	Restricted + betaine	2 w	3.59 y	700–1100	Abnormal (3.5 and 4.5 y)	Normal
62	Normal	Birth	< 5 m	461 (1)		
	Normal	1 y	2.1 y	860 ± 424 (3)		Developmental delay; autistic action (2.2 y)
	Restricted	2.1 y	2.4 y	7 (1)	Normal (2.3 y)	Improved (2.4 y)
	Normal	2.4 y	4.3 y	741 ± 71 (2)		IQ = 108 (3.3 y)
						Normal (4.3 y)
63	Normal	Birth	1 m	1105 ± 235 (2)		
	Restricted	1 m	1 y	570 (1)	Abnormal (0.8 y)	
	Normal	1 y	2.9 y	905 ± 99 (3)	Worse (1.5 y)	No neurological signs (2.9 y)
Patients treated with AdoMet supplementation only						
4	Normal	Birth	11 y	600–1400 (2)	Abnormal (11 y)	Headaches, nystagmus, dysdiadochokinesis, increased tendon reflexes (11 y)
	AdoMet, 400 mg bid	11 y	12 y		Normal (12 y)	
	AdoMet, 200 mg bid	17 y	32 y	565 ± 139 (17)		Normal baby daughter (30 y)
22	Normal	Birth	4.7 y	1005–1676 (2)	Abnormal (4.7 y)	IQ 49 (3.7 y)
	AdoMet, 400–800 mg bid	4.7	7.4 y	1528 (1)	Normal (6.4 y)	IQ improved to 69 (5.4y); awake EEG markedly improved (6.3 y); IQ 64 (7.4 y)
35	Normal	Birth	3 m	1620 ± 107 (2)		
	?	3 m	1.2 y	1159 (1)	Abnormal (1.2 y)	
	AdoMet 100–200 mg bid	1.3 y	1.8 y	759 ± 278 (2)	Improved overall (1.8 y)	
50	Normal	Birth	<4.3 y	878 ± 136 (3)	Abnormal (4.3 y)	
	AdoMet, 200 mg bid	4.4 y	4.5 y			
	AdoMet, 400 mg bid	4.5 y	4.9 y			Behavior considerably improved
51	Normal	Birth	3.3 y	641 ± 74 (2)	Abnormal	
	AdoMet, 400 mg bid	4.7 y				Not available
Patients who had periods of dietary restriction and AdoMet supplementation						
7	Normal	Birth	0.5 m	1445 ± 310 (2)		
	Restricted	1 m	4 m	516 ± 192 (5)		
	Normal	4 m	6 m	1138 (1)		
	Restricted	6 m	9 m	1451 (1)	Normal (9 m)	Developmental delays
	Normal	9 m	14 y	1457 ± 322 (10)	Abnormal (4.2 y)	Impaired function; fine tremor
	AdoMet, 200 mg/d	14.1 y				Brief trial of AdoMet . Attempts to assess efficacy failed due to loss of follow-up. The supplement was stopped by the family who considered there had not been noticeable clinical improvement.
34	Normal	Birth	5.5 m			
	Restricted + AdoMet	5.5 m	8.3m			Improved clinically on combined methionine restriction and AdoMet supplemenation, but details not available.
41	Normal	Birth	0.2 y	919 ± 795 (4)		
	Restricted	0.2 y	6.7 y	313 ± 226 (9)	Abnormal (0.2 y)	
	Restricted + AdoMet	6.7 y	7.6 y	455 ± 104 (5)	Normal (7.1 y)	Appetite improved (7.1 y)
45	Normal	Birth	1.8 m	760 ± 357 (2)		
	Normal	1.8 m	4.1 y	901 ± 163 (4)	Abnormal (4 y)	Physical and neurologic examinations normal (4 y)
	Restricted + AdoMet	4.1	4.7	468 ± 120 (4)	Improved (4.5 y)	

*Diet classified as methionine restriction only if continued for > 0.4 years and decreased plasma methionine by > 90 μM

w = weeks

m = months

y = years

**Table 4:** Histories of patient with possible abnormal CNS findings

Patient 1: At age 24 y, patient 1 had normal intelligence and no signs of neurological abnormalities. However, in an MRI at 20 y, signal from gray substance appeared well differentiated in first “echo” from the T2 sequences, but was inverted in the second echo, principally in peripheral regions. There was also a heterogeneous hyposignal from the posterior part of the central gray nuclei.
4: At age 11 y patient 4 attended normal school, but developed severe headaches. Nystagmus, dysdiadochokinesis and increased tendon reflexes were found. An MRI showed delayed myelination in all but the internal capsule and brainstem. On AdoMet supplementation symptoms and signs completely resolved and MRI showed restoration of normal myelination
Patient 5: At age 14 y this patient was considered slow relative to the rest of her family and to have learning disability. At age 7.5 years she had, on the Wechsler Intelligence Scale for Children-Revised, a verbal score of 88 and a performance score of 84. An MRI at age 13 y was normal, including normal myelination.
Patient 7: Patient 7 was on methionine restriction between 1 month and 4 months, and again from 6 months to 9 months. An MRI at that time was normal and a normal diet was restarted. Early gross motor milestones were delayed. Although neurologically normal at age 4 y, an MRI showed diffusely abnormal white matter and patchy basal ganglia bilaterally and abnormally high signal in the inferior aspect and inferior midbrain. At age 7 y the boy was in special class with memory and learning disabilities. MRI was stable at age 14 y. A short trial of AdoMet was stopped by the family because they did not notice improvement in clinical status. At age 17.8 years there was notable impaired function and a fine tremor.
Patient 8: At age 7 y this girl had tremor and increased tone in her right arm with dystonia and dysmetria. An MRI at 9 y showed decreased T1 signal and increased proton density in the left posterior putamen abutting the posterior limb of the internal capsule. At age 11 y, myelination was arrested to about 1½ year level.
Patient 9: At age 6 y patient 9 had learning disability. On a Stanford-Binet Intelligence test he scored 65 (1^st^ percentile), but an MRI was entirely normal.
Patient 13: An MRI of this girl at 8 y showed patchy alterations of white matter. At age 16 she required extra time to complete high-school tasks and was given an “Individualized Education Program”.
Patient 20: An MRI at age 10 y showed white matter changes.
Patient 22: At age 2.8 y patient 22 had febrile seizures with clonic jerks of the right arm and bilateral clonic seizures. An MRI showed myelination arrest in the central white matter, putamen, and globus pallidus. An MRI at 4.7 y showed delayed myelination, and supplemental AdoMet was started. At age 5.4 y her score on an intelligence test improved to 69. An MRI at 6.4 y showed normal myelination.
Patient 29: As early as age 2 y this boy was suspected to have mild mental retardation. At age 12 y he started to have concentration difficulties in school and his performance worsened. At age 13 y full scale IQ was 73 (3.6 percentile) and an MRI showed normal gray-white matter differentiation in T1 weighted sequence and overall mild hyperintensity of the subcortical white matter in T2 weighted sequence, suspicious of hypomyelinization.
Patient 30: From age 3 weeks patient 30 was on dietary methionine restriction and betaine supplementation. He was neurologically unremarkable with an IQ of 121 at age 3.5 y, but an MRI at 3.8 y showed diffuse T1 and T2 prolongation in the subcortical areas extending to deep white matter, with relative sparing of the corticospinal tracts, corpus callosum, optic radiations, ventral brain stem, and cerebellar white matter. There were symmetric lesions in the dorsal brain stem from the red nucleus to the olivary nucleus areas. Methionine restriction and betaine were discontinued at age 4 y, and at age 5 y MRI findings improved, although patchy foci of T1 and T2 shortening were still observed in the white matter [37]. At age 7 y the delay of myelination had disappeared [41].
Patient 31: This sib of #30 was on dietary methionine restriction from age 3 months to 1.8 years. An MRI at 0.8 y showed delayed myelination of white matter with T1 and T2 prolongation in the symmetric tegmental tract. At 3 y his development was normal and neurological examination unremarkable, but MRI showed delayed myelination in white matter with symmetric lesions in the dorsal brain stem [41].
Patient 32: Patient 32 was on methionine restriction from an early age. An MRI at 5 y showed delayed myelination equivalent to that in normal children’s at age 6 months. Methionine restriction was discontinued. An MRI at age 5.5 y was unchanged [36]. At age 9.4 y his IQ was 108, physical and neurological examinations were normal, but myelination was the same as that at 5.5 y.
Patient 34: This child of consanguineous parents had a large head, facial dysmorphia, severely retarded, slow and infrequent movements of the body, head and eyes with suspected central visual impairment. After start of methionine restriction and AdoMet supplementation at about 5.5 months of age, he was said by age 8.3 months to have improved, but details are not available.
Patient 35: An MRI, performed at age at 1.2 y because MAT I/III deficiency with severe hypermethioninemia had been established, showed bilaterally symmetrical restricted diffusion in the commissural fibers of the corpus callosum, anterior commissure, internal capsule, and globus pallidus. Spectroscopy demonstrated a diminished NAA peak. MRI at 1.8 y after 6 months of AdoMet administration, showed improvement in areas of restricted diffusion in the corpus callosum, anterior commissure, globi pallid, and white matter. The right anterior commissure was the only area where restricted diffusion was possibly slightly increased.
Patient 36. This identical twin of patient 37, was born prematurely at 32 weeks gestation. At age 2.3 y each twin had developmental delays, especially of speech, but an MRI of brain of one twin was normal.
Patient 37: See identical twin, patient 36.
Patient 38: This daughter of consanguineous parents and sib of patient 39 was noted at age 3 y to have developmental delay. At age 5 y she had myoclonic epilepsy treated with valproic acid. At age 11.1 y she was perfectly healthy apart from learning difficulties for which she was in a special class at grade 2 level. At age 12.8 y she was in 4^th^ grade rather than 7^th^. An MRI showed prolonged relaxation time and shortened apparent diffusion coefficient
Patient 39: An MRI at 5.2 y showed: prolonged relaxation time, and 2shortened apparent diffusion coefficient in this boy. At age 10.5 years he was in 3^rd^ grade rather than 5^th^, but otherwise well.
Patient 41: An MRI at 0.24 y showed: abnormal symmetric T2 hyperintensity along the cortical spinal tract from the perirolandic white matter through the centrum semiovale, post limb internal capsule, cerebral peduncles, anterior pons and medulla. Also present were symmetric abnormal signal within the dorsal brainstem tegmental tract, extending into the cerebellum and along the hippocampal formations and within the fornix. Within these areas there was restricted diffusion and diffuse swelling. The patient was started on methionine restriction which has continued to present. AdoMet supplementation was started at age 6.7 y. An MRI at 7.1 y was normal and the parents think AdoMet has been very helpful.
Patient 42: An MRI of this boy at 1.2 y showed bilateral symmetrical areas of hyperintense T2-weighted images in white matter at the level of temporal-insular and temporal-mesial, deep parietal, ponto-mesencephalic, dorsal and cerebral peduncles with involvement of the structures above and the capsular lenticular corpus callosum. ADC values were reduced to diffusion. At age 1.5 y he was noted to have speech delay.
Patient 43: This boy was on methionine restriction from age 0.8–3.2 y. At 3.3 y a neuropsychological evaluation found a developmental age of 27 months and IQ’s of 78 on performance and 57 on reasoning. The diet was restarted. An MRI at 3.8 y showed delayed myelination peripherally throughout. On FLAIR examination there were multiple periventricular focal white matter hyperintensities.
Patient 45: At age 4 y physical and neurological examinations and physiotherapeutic testing of patient 45 were normal, but an MRI showed white matter changes and delayed myelination. At age 4.1 y she started protein restriction and supplemental AdoMet. At age 4.5 y physical and neurological examinations and physiotherapeutic testing continued to be normal. An MRI showed normal white matter and delayed myelination.
Patient 46: This woman was on methionine restriction from age 0.1–4.0 y, then on a normal diet. Her IQ by WISC-R at age 9 y was 99 [15, 46]. An MRI at age 30 y showed T1 and T2 prolongation in the deep white matter, but physical and mental development have been entirely normal.
Patient 47: A dietary history is not available for this patient. At age 9 y she had dysdiadochokinesia and MRI showed hypomyelination. At age 10 she had normal cognition and an MRI at age 17 y was almost normal.
Patient 50: An MRI of patient 50 at 4.3 y showed: white matter changes, in particular restricted diffusion in the area of the genu of the corpus callosum. There were no changes in T1 or T2. Supplementation with AdoMet, 200 mg/d was started at age 4.4 y and the dose increased to 400 mg/d at 4.5 y. The patient’s behavior with regard to aggression and hyperactivity were improved at 4.9 y.
Patient 51: The behavior of this younger sister of patient 50 deteriorated by age 3.7 y and supplementation with AdoMet was started at 200 mg/d. Outcome not know to date.
Patient 56: This woman was ascertained and genotyped at age 38 y as a result of having given birth to two children with transient hypermethioninemia on newborn screening. She had never been on methionine restriction. She received average grades throughout school and has an associate degree. She has had hand tremors since childhood and is affected with depression. Physical examination showed increased deep tendon reflexes, tremors, dysmetria and dysdiadochokinesis.
Patient 60: Despite her normal development until age 1.4 y, an MRI showed extensive abnormal signal in the white matter of the corpus callosum and of the anterior commissure. Neuroimaging at age 2 years showed similar results [40]. At age 5.6 years she showed possible mild neuropsychotic delay and hyperreflexia.
Patient 61: At early age (3 weeks) this boy was treated with methionine restriction and betaine because of mild hyperhomocysteinemia. At age 2.5 years pyridoxine was added. Treatment was discontinued at age 3.9 years. Brain demyelination was revealed at age 3.5 years and confirmed at 4.5 years. At age 5.5 no neurological symptoms were reported.
Patient 62: At age 2.1 years developmental delay and autistic action of this boy led to the start of dietary methionine restriction. An MRI at age 2.3 y was normal and the diet was discontinued at age 2.4 y. IQ is 108 at age 3.3 y. At age 4.3 y the boy was developing normally and attending kindergarten.
Patient 63: At age 10 months an MRI of patient 63 showed diffuse abnormal lesions in the white matter indicative of delayed myelination. T2-weighted images showed no myelination in the external capsules or anterior limbs of the internal capsules, but lack of T1-weighted images made evaluation difficult. At 18 months the abnormal lesions in the white matter were more extensive. However, at age 2.9 y, there were no neurological signs.

### Possible criteria to distinguish between those with and without CNS problem (Tables 1 and 2)

#### MAT1A genotypes

*MAT1A* genotypes are not clearly predictive. Patients with homozygous truncating mutations with presumably no residual MAT I/III activity are found among those without CNS problems (patients 2 and 3), and most of the patients with CNS problems have only missense mutations which, when expressed in *E. coli*, had some residual MAT activities.

#### Plasma methionine concentrations

**Fig. 1:**
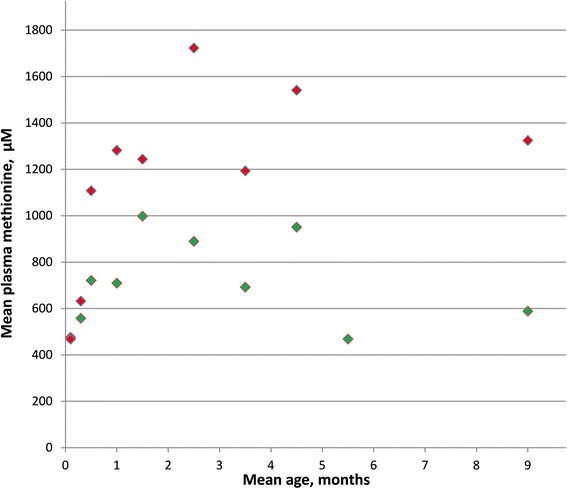
Mean plasma methionine values of untreated patients in the first months of life are plotted against mean ages at which samples were drawn. Red markers indicate patients with evidence of CNS abnormalities; green markers, patients without such evidence

**Fig 2:**
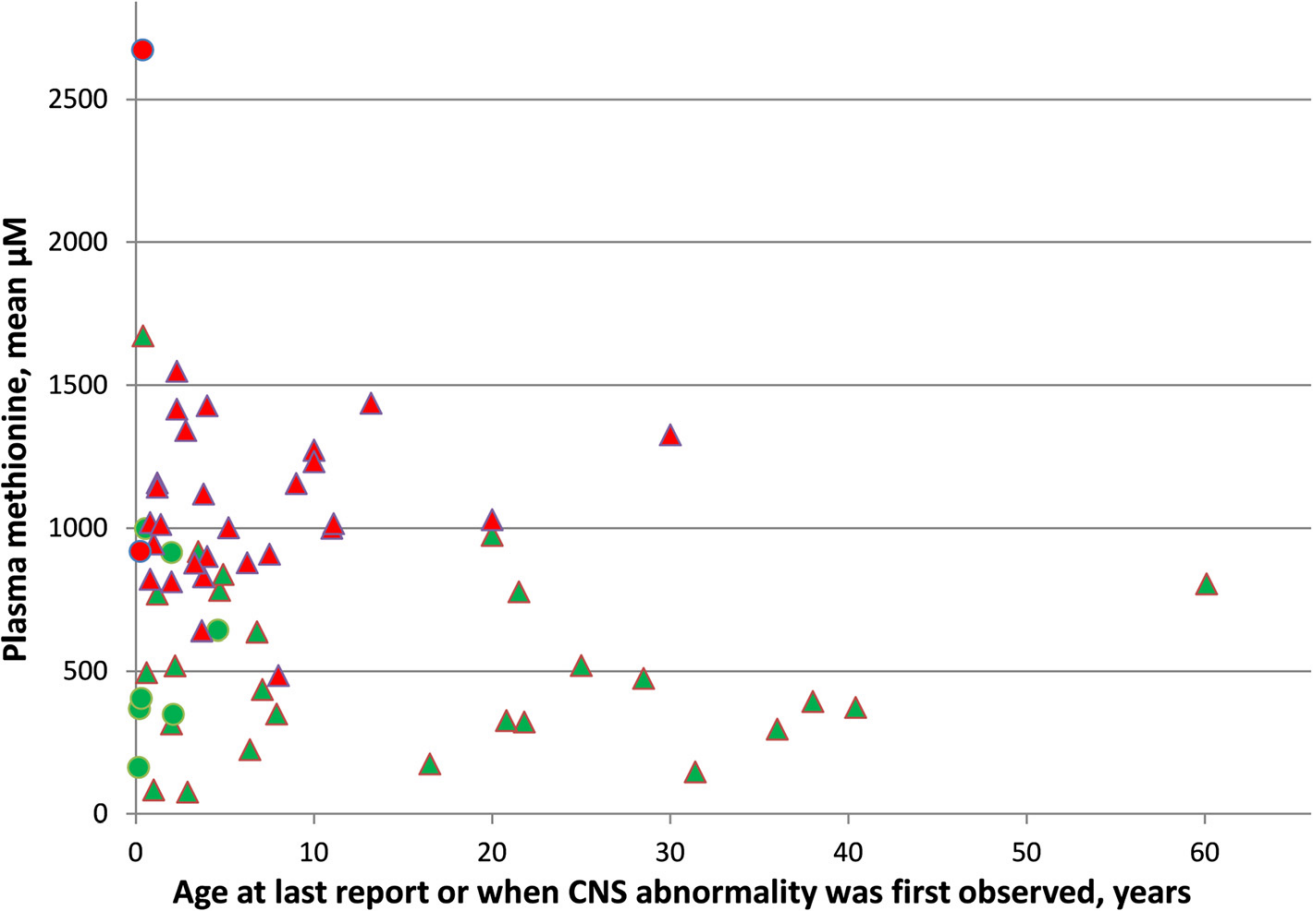
Plasma methionine concentrations for patients on normal diets without (green) or with (red) evidence of CNS abnormalities. Green values are plotted against age at last report; red values, against age at which CNS abnormality was first found. Triangles indicate mean methionine values at ages ≥ 5 months; circles indicate mean methionine values at ages < 5 months (if values at older ages are not available; and plotted against age at last report)

For untreated patients for whom more than one value for plasma methionine during the first few months of life were available, values often varied relatively widely (Fig. 1). After that time methionine concentrations tended to be less variable for a given patient. As shown in Fig. 2, on normal diets mean methionine values for patients with evidence of CNS abnormalities were usually higher than were those for patients without evidence of such abnormalities. Those with mean values above 800 μM almost always have CNS abnormalities, whereas those with means less than 800 μM usually do not. In the total group (all ages) three patients with CNS abnormalities were below 800 μM and six patients without CNS abnormalies were above 800 μM. The respective means (± SD) for these two groups were 1073 μM ± 233 and 485 μM ± 276.

#### Plasma AdoMet concentrations

**Fig. 3:**
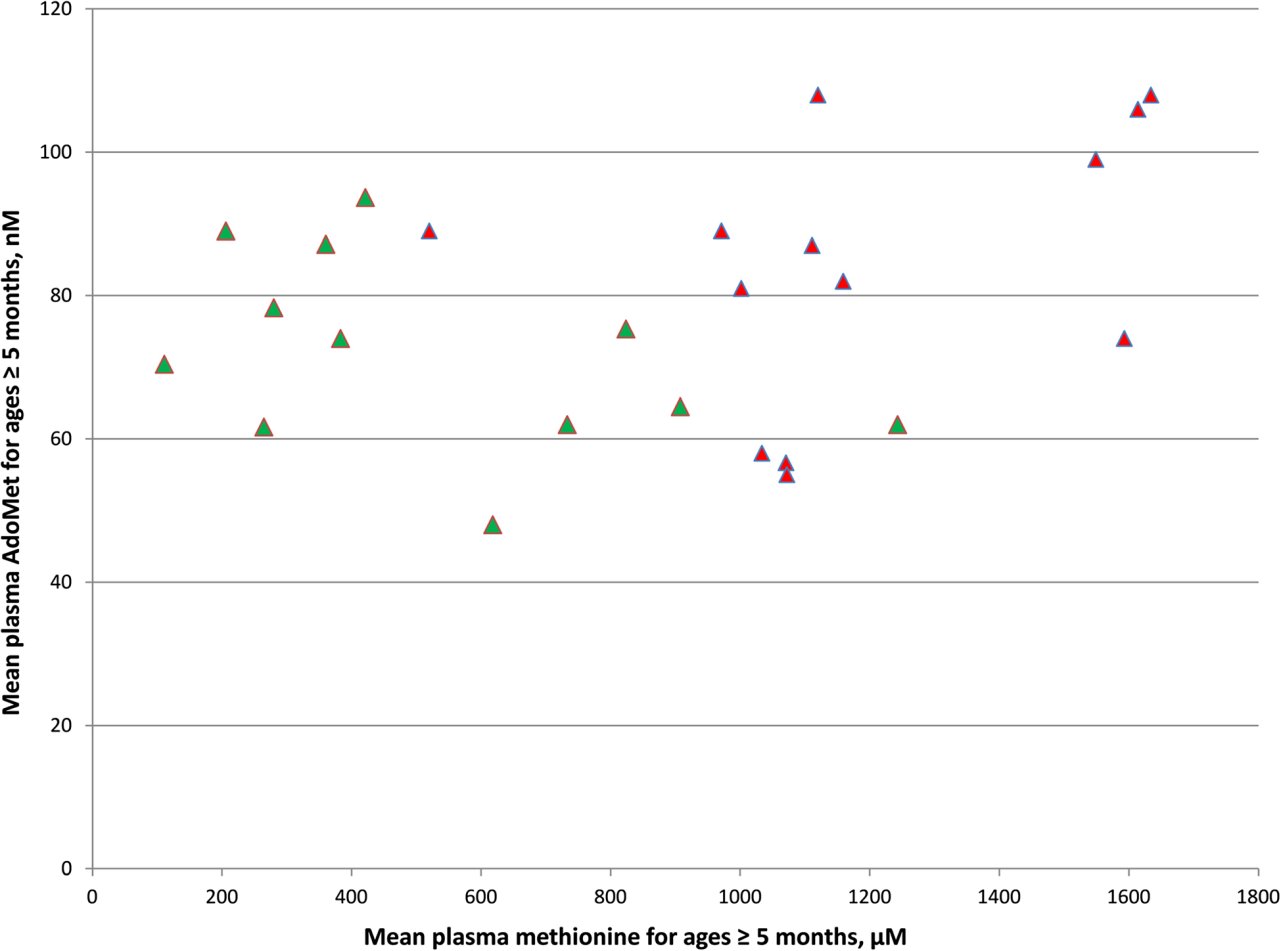
Plasma AdoMet as a function of plasma methionine. Symbols are the same as those used in Fig. 2. For each patient the mean plasma methionine concentration on normal diets at ages ≥ 5 months is plotted against the mean plasma AdoMet concentration on normal diets at ages ≥ 5 months. Patients without and with evidence of CNS abnormalities are shown, respectively, by red or green markers

Data for the available concentrations of plasma AdoMet at age ≥ 5 months are shown in Fig. 3. Although, as already shown in Fig. 2, most patients with evidence of CNS abnormalities have higher plasma methionines, there is no trend for a difference in the AdoMet concentrations between those with and without such evidence, and the mean values (± SD) were, respectively 84 nM ± 19 and 72 nM ± 13.

#### Plasma total homocysteine (tHcy) values

**Fig. 4:**
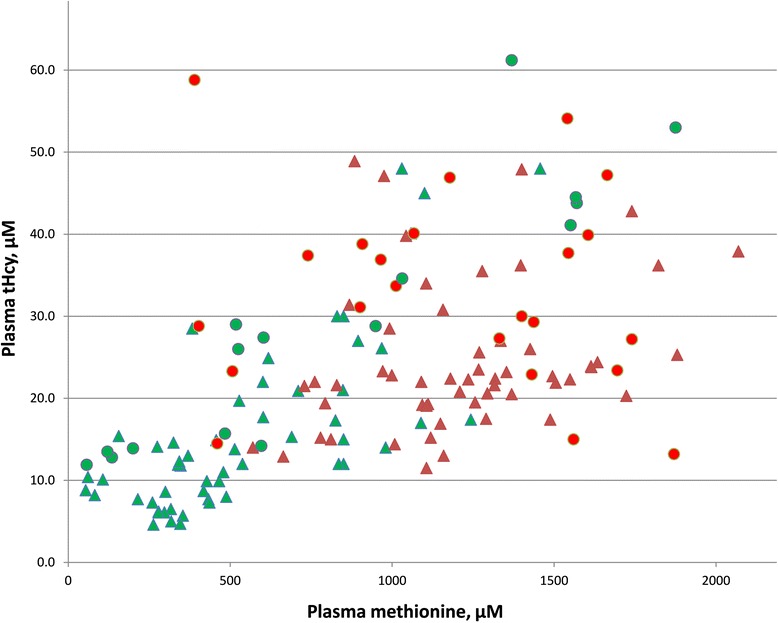
Values for plasma methionine and tHcy are plotted for individual samples from patients on normal diets. Red and green triangles and circles are used as in Fig. 2 to indicate either presence or absence of CNS abnormalities and ages either above or below 5 months

Figure 4 shows values of plasma methionine and tHcy for the individual samples in which both of these were assayed. To provide more indication of the range of values encountered at young ages, points are plotted for samples drawn either before (circles) or after (triangles) age 5 months. As plasma methionine rises, tHcy tends to rise also, so that patients with evidence of CNS abnormalities tend to have higher levels of tHcy. However at the higher methionine concentrations there is no apparent difference in the tHcy values between those with and without CNS abnormalities, indicating the response to methionine elevation is about the same in the two groups.

#### Plasma cystathionine values

Among the few patients for whom sensitive serial assays of cystathionine were performed at early ages cystathionines were mildly elevated at 16 days, 21 days, 38 days and 1.5 months with values, respectively of 776, 1196, 1280, and 1001 nM (adult reference range 44–342 nM). Values had fallen to 566 and 670 nM by 11 months and thereafter were, at most, only slightly above the reference range (data not shown in Tables).

### Liver function studies

The results of liver function studies are available for 16/32 patients without and 14/32 of those with evidence of CNS abnormalities. Included are assays (in some patient multiple times) of ALT and AST, total bilirubin, alkaline phosphatase, albumin, total plasma protein, and gamma glutamyltranspeptidase. No instances of hepatic malfunction have been detected by these studies (data not shown in Tables).

### Pregnancy outcomes

Information is available on 15 pregnancies among six of the present patients:

- *Patient G1* had four pregnancies. Because the patient’s plasma choline was below the reference range, and because brain damage occurs in fetuses of experimental animal mothers fed low choline diets [45], she was advised after gestation week 17 in three of these pregnancies to ingest two eggs daily to provide about 630 mg choline/day. Three normal children were born who have continued to develop normally mentally and physically. In the fourth pregnancy there were no overt complications but sonography at ten weeks disclosed fetal arrest and the pregnancy was terminated two weeks later [46].
- *Patient G4* took 600 mg choline/day during most of her initial pregnancy and produced a normal baby. During a second pregnancy she took 2 eggs/day from 18 weeks gestation, and then changed to choline capsules, 600 mg/d during the remainder of the pregnancy. A normal child was born who continued to be normal at last investigation at age 6 months.
- *Patient 3* had been temporarily lost to follow-up, but was relocated and found to have produced a normal baby without any specific treatment due to her MAT I/III deficiency.
- *Patient 5* had also been lost to follow-up after childhood, but was found again when she produced a baby that was flagged during NBS for elevated methionine that turned out to be transient [47].
- *Patient 4* produced a normal baby after a pregnancy during which she continued her usual dose of AdoMet, 200 mg bid.
- *Patient 56* was ascertained at age 38 with elevated methionine and tHcy because she had given birth to two children with transient hypermethioninemia (NBS methionines of 552 and 554 μM, falling to 94 μM on day 5 or 42 μM on day 4 of life). Her history revealed that she had had six pregnancies with one lost at 4 weeks, a stillborn at 16 weeks, two blighted ovums and another 2 with transient hypermethioninemia.

### Treatment outcomes

#### Vitamin B6

Because of the mild elevations of tHcy often encountered in *MAT1A* deficient patients, 6 of the 32 without and 7 of the 32 with evidence of CNS abnormalities (data not shown in Tables) have been given brief trials of high doses of vitamin B6. In almost all cases there were no significant effects. One patient (#36) did experience a marked decrease in plasma methionine during an initial period of B6 treatment, but that response did not occur during a second trial nor in her identical twin sister (#37). In addition, even some adverse events were reported in patients using high doses of B6 [48]. One MAT I/III deficient infant (#7) became apneic and required respiratory support shortly after starting a dose of pyridoxine of 500 mg/day [49], and Tada et al. reported that pyridoxine treatment may have worsened the MRI and neurological abnormalities in their patient (#30) [36]. Taken together, high doses of vitamin B6 should not be used in patients with MAT I/III deficiency.

#### Dietary methionine restriction

Methionine restriction has been used for 8/32 patients without CNS abnormalities and 16/32 patients with CNS abnormalities, starting at various ages and continued for a range of times. Details for each such patient are reported in Table 3 (see supplementary data), together with the mean plasma methionine concentrations before, during, and after restriction and very brief comments describing if and when MRI or clinical abnormalities were observed. For each patient with evidence of CNS abnormalities more detailed clinical histories and the reports of MRI findings are presented in Table 4 (see supplementary data). The pros and cons of using dietary restriction are described in more detail in the “Discussion” [sections 4.4.1 and 4.4.2].

#### AdoMet supplementation

AdoMet supplementation has been used for nine patients, each of whom had abnormal CNS findings. AdoMet was given during periods of methionine restriction in four of these nine patients. Summaries of these trials are listed in Table 3 with more details in Table 4. AdoMet supplementation is also discussed in the “Discussion” [section 5.2.1].

## Discussion

Sufficient information has been gathered during the present survey to permit discussion of several important questions about MAT I/III deficiency:

### Diagnosis

The possibility of MAT I/III deficiency usually arises with the finding of elevated plasma methionine without the marked elevations of tHcy that occur in CBS deficiency [50]. However, uncertainty may arise because, as shown in Fig. 4, MAT I/III deficient patients tend to have mild elevations of tHcy as their methionine levels rise. Assay of plasma AdoMet then becomes a useful next step, since plasma AdoMet is elevated in CBS deficiency (median 488 nM; 25–75 percentile, 155–868) [51], but is usually not elevated in samples from MAT I/III deficient individuals drawn at age of more that 3–4 months (Fig. 3). However, the few MAT I/III deficient patients among those under discussion who had early samples assayed for AdoMet often had very mild elevations of AdoMet (as high as 152 nM), and similar or slightly higher elevations (up to 220 nM) have been found in early samples from patients with one of the *MAT1A* mutations (e.g., R264H) that cause slight to moderate hypermethioninemia in heterozygotes. These rises do not persist beyond about age three months, and may perhaps be due to incomplete turning off of the expression of MAT II that occurs after birth. Thus, when investigating a child who at an early age has elevated methionine and, at most, slight elevations of tHcy and AdoMet, further evidence as to whether that child is a heterozygote, homozygote, or compound heterozygote for *MAT1A* mutation(s), may be obtained by assaying plasma methionine in both parents. If the child is a heterozygote for a dominant mutation one parent will also have at least a minimal elevation of plasma methionine. Strong evidence indicates that such heterozygotes will be clinically unaffected [30, 31]. If neither parent is hypermethioninemic, to pin down the diagnosis the *MAT1A* gene may be sequenced so that further monitoring and/or trials of therapy can take place if the child turns out to be a homozygote or compound heterozygote for *MAT1A* mutation(s).

Another finding is elevated cystathionine in samples of patients of young age, which may even be used as an additional marker to distinguish MATI/III deficiency from CBS deficiency [52]. Again, this elevation is transient and may probably be attributed to incomplete expression of cystathionine gamma-lyase, which activity is not expressed in fetal liver [53].

### Liver function and hepatocellular carcinoma

*MAT1A* knocked out mice develop oxidative stress and nonalcoholic steatohepatitis by 8 months [54] and, a high incidence of hepatocellular carcinoma by 18 months [55]. Liver histology was studied in a few early MAT I/III-deficient patients who had liver biopsies performed to assay MAT activity before *MAT1A* genotyping became available. Light microscopy was usually normal (patients G1, G3 [17]; #2 [19]; #1 [14]; and #46 [15]), but electron microscopy showed a variety of abnormalities. Mitochondria were either normal (#2 [19]) or had breaks in their outer membranes (G1 [17]), or elongated (G2 [17]) or abnormal (#1 [14]) shapes. Smooth endoplasmic reticulum was increased in amount (G1 [17]; #2 [19]; and #46 [15]); rough endoplasmic reticulum, decreased (G1 [17]; #46 [15]). Increased pericellular collagen fibers were present in the space of Disse (G1; G2 [17]; and #46 [15]), and residual pigment in lysosomes of two patient (G3; G4 [17]). Together, these findings raise concern about liver function in MAT I/III-deficient patients. To date hepatocellular carcinoma has not been found in any of the patients under discussion. Although most are still relatively young, at last report patient #2 was 60, four patients were ages 32 to 40; and nine were in their twenties. Taken together with the fact that the liver function tests in the 30/64 patients so investigated were normal (including several (G1, G2, G4, #2, #46) who had hepatic electron microscopic changes) (data not shown in Tables), these results are encouraging with regard to liver function and the chances of developing hepatic malignancies.

### Pregnancy outcomes

The fact that five of the patients produced eight normal babies from nine pregnancies is encouraging. However, one patient had only two normal babies as a result of six pregnancies. Further experience will be needed to fully evaluate the effects of maternal MAT I/III-deficiency on pregnancy.

### CNS abnormalities

Of the 64 patients under discussion, 32 have been classified as not having evidence of CNS abnormalities, whereas 32 have been classified as having such evidence. It should be noted, however, that whether a given patient is assigned to the group of those without or with CNS problems is subject to several limitations: (a) several of the patients without evidence of CNS abnormalities are, as yet, very young (see Table 1 and Fig. 2). CNS problems may arise as they age. (b) The classification is based most often upon the reported presence of MRI abnormalities, and there may be some ascertainment bias with regard to carrying out MRI studies. Although the majority of patients in both groups were ascertained by screening of newborns or family members (31/32 and 27/32, respectively), among those without evidence of CNS abnormalities MRI’s were done on only 6/32 and were in all cases normal, whereas among those classified as having CNS abnormalities 28/32 had MRI studies with 4 being normal. In the rest 24 being abnormal, 5 became normal and 2 reported as improving. Thus it is possible that had more of the group without evidence of CNS abnormalities had MRI’s, more abnormalities would have been found. (c) The grounds for classifying a few of the patients as having CNS abnormalities may be weak or not even causally related to MAT I/III deficiency. The most questionable cases are patient 5 who was considered slow at learning and had Wechsler Intelligence Scale scores of verbal 86 and performance 84; patient 13 who was slow at school: and, perhaps, patient 9 who had an IQ of 65 (1^st^ percentile). In spite of these limitations, as already mentioned in the “Results” section, the scatter diagram of patients with and without CNS abnormalities against their mean plasma methionines (Fig. 2) suggests stratification: those without CNS problems usually have methionines below 800 μM; those with CNS problems have plasma methionines of 800 to almost 1500 μM.

#### Has dietary methionine restriction, the treatment most often used, been harmful?

There is concern that, if some residual MAT I/III activity is still present, lowering methionine concentrations might further limit the flux due to that residual activity, as well as the flux through MAT II, thereby further decreasing the availability of AdoMet and aggravating the possibility of developing CNS problems [50]. The present study produced results for eight patients, each of whom has had some period of methionine restriction, who remained free of evidence of CNS abnormalities (see first section of Table 3). Five of these had only missense mutations; patient 48 had one missense and one truncating mutation; patient 58 was not sequenced. The methionine restriction used for these individuals did no apparent harm. Patient 13 is one in whom restriction may possibly have had adverse effects. Although his methionine on normal diets at ages was 485 μM, after methionine restriction from age 1.7 to 10 months with a mean level of 193 μM he was found at age 8 to have an abnormal MRI and was judged to be slow at school.

#### Has methionine restriction been beneficial?

Evidence as to the usefulness of methionine restriction is limited and prevents drawing any firm conclusions. Findings that might be considered strongly convincing of benefit would be for example an individual with methionine values of > 900 μM at ≥ 5 months on normal diets, who after using a methionine restricted diet remained free of CNS abnormalities, but such informative cases are not available. For the eight patients without evidence of CNS abnormalities who had been on dietary restriction (Table 3), mean methionine values on normal diets at ages ≥ 5 months ranged from 175 to 777 μM (mean 388 ± 208), so there is a likelihood that they would not have had CNS abnormalities even if they had not been on methionine restriction. The findings for several patients, however, are at least suggestive of benefit: patient 20 remained normal until age 5.4 years while on methionine restriction that kept his methionine down to 684 μM, but developed MRI abnormalities after diet was discontinued and methionine climbed to 1298 μM. Patient 1 (during) and patient 46 (after) methionine restriction are normally intelligent at ages 20.7 and 34 years, although each has MRI abnormalities. Patient 13 developed abnormalities after stopping methionine restriction. Patient 62’s behavior improved on restriction and patient 63’s MRI worsened after stopping restriction.

## Pathophysiology

As just detailed, judged by the data on outcomes, the evidence available as to optimal treatment of the patients in question is unclear. An alternative means to try to find the optimal treatment might be to consider the pathophysiology of the metabolic abnormalities in MAT I/III deficiency. There is strong evidence of stratification between the presence or absence of CNS problems and mean methionine concentrations. This could indicate toxicity of very high methionine concentrations itself, deficiency of AdoMet due to more severe loss of MAT I/III activity, the rises in tHcy, or some combination of more than one of these factors. Details of each of these alternatives are discussed below.

### Methionine elevation

#### High plasma methionine and brain edema

The MAT I/III deficient patient of Tada et al. (#30) developed brain edema while on betaine (and B6) treatment resulting in methionine concentrations between 960 and 1560 μM [36]. Braverman et al. [52] reviewed four cases with edema and severe elevations of plasma methionine. Brain edema developed in two CBS deficient patients on betaine with methionine of 1190 and 3000 μM. Because betaine is also an intracellular osmolyte, its high levels may have contributed to the development of edema. In the same report Braverman et al. reviewed two normal infants having edema after an extreme high methionine intake resulting in plasma methionines of 2154 and 6830 μM.

Taken together very severe elevations of methionine (above 2000 μM) may induce edema, whereas the use of betaine may already lead to edema if plasma methionine is above 1000 μM. Although edema is only reported in patients with MAT I/III deficiency if using betaine, it can not be ruled out that a more subtle inability of cells to pump water adequately may contribute to CNS problems.

#### Might methionine inhibit some methyltransferase activity necessary for myelination or another vital CNS function?

Methyl transfer reactions definitely occur in brain [56], but their quantitative extent has not been established. A review of the literature turned up no instances of methionine inhibition of an AdoMet-dependent methyl transfer reaction.

#### Might some break-down product of methionine play a role in CNS problems?

Dever and Elfarra [57] pointed out that products of the methionine transamination pathway and/or sulfoxidation should be considered as a potential cause of CNS problems. It is well established that patients with methionine elevations now known to be due to MAT I/III deficiency utilize transamination when plasma methionine is above about 350 μM [58]. Evidence on transamination or sulfoxidation products has not been collected during the present survey. We do know that Patient 2, a homozygote for a truncating mutation, presented with bad breath odor due to dimethylsulfide formed by the transamination pathway [20], but that he remains clinically well at age 60 years. Another argument against the transamination and/or sulfoxidation products hypothesis is that it would not explain the beneficial effect of AdoMet supplementation, for which there is relatively strong evidence (as mentioned in sections 5.1.5 and 5.2.1).

#### Might very high methionine compete for transport of other amino acids across the blood–brain-barrier so severely as to cause damage?

Methionine is transported across the blood–brain-barrier by the “large neutral amino acid” (LNAA) system, as are nine other amino acids. Phenylalanine has the lowest Km, 11 μM, for this system; methionine is the fourth lowest, 40 μM [59]. The system normally functions near saturation with LNAA’s, so that if one of them rises above its normal plasma level, to some extent, it will compete with the other LNAA’s and inhibit their transport across the blood–brain-barrier [59]. One hypothesis to explain the CNS damage of phenylketonuria (PKU) is that competition by phenylalanine for LNAA transport accounts for the adverse effects [60]. Until recently there was an international consensus that phenylalanine levels of < 360 μM are not harmful and do not need treatment. More recent evidence has shown that even phenylalanine levels of as high as 800 μM are either clinically benign [61], or lead only to MRI changes of uncertain significance [62]. Because the Km of methionine for the LNAA transporter is 40/11 = 3.6-fold higher than that of phenylalanine, it seems that the extent of competitive inhibition of LNAA uptake by methionine concentrations of as much as 3.6 × 360 μM = 1296 μM are unlikely to be harmful, whereas concentrations above 1296 μM and up to 3.6 × 800 = 2880 μM might bring about MRI changes.

#### Might high plasma methionine inhibit the transport of AdoMet into brain?

Chishty et al. describe the carrier mediated transport of AdoMet to the blood–brain barrier by system(s) that transport adenosine, but not methionine [63]. Therefore, it seems unlikely that methionine interferes with transport of AdoMet across the blood–brain barrier. CSF AdoMet concentrations without AdoMet supplementation are available for three patients, each of whom had CNS problems: two, patient 4 [64] and patient 29 [44], had somewhat low levels; one (patient 43) had a normal level. It is noteworthy that the concentration of AdoMet in CSF may not accurately reflect the brain tissue concentration. For example, there was no significant relationship between AdoMet concentrations in CSF and those in brain after treatment of pigs with nitrous oxide which dramatically changed the AdoMet/AdoHcy ratio [65]. Metz also discussed this discrepancy in his review article [66]. In spite of these limitations, it appears that there is no strong evidence that high plasma methionine interferes with delivery of AdoMet from plasma to brain.

#### Might very high methionine somehow decrease the AdoMet concentration of the brain?

One possibility is that methionine inhibits MAT II activity. However, studies of the kinetic properties of MAT II do not mention methionine inhibition of this activity [67], even when brain MAT II was studied [68]. Another possibility is that high methionine might lead to decreases in the amount, rather than the activity, of MAT II. Such an effect has been reported in both human hepatoma [69] and neuroblastoma cells [70], but not in H35 or cos7 cells [71]. However the effect in hepatoma cells depended upon synthesis of AdoMet, so it seems unlikely to occur in brain in cases of MAT I/III deficiency. Direct evidence of the effect of high methionine on brain AdoMet concentrations has been reported by Young and Shalchi, who assayed AdoMet in rat brains after administration of increasing doses of methionine and found a tendency for brain AdoMet concentrations to decrease with the higher doses of methionine [72]. If the brain AdoMet levels in the MAT I/III-deficient patients follow the tendency reported by Young and Shalchi to decrease in the presence of especially high methionine, and fall below normal with the highest plasma methionines (a proposition that needs to be tested, at least on experimental animals) it would then be plausible to suggest that the CNS problems of MAT I/III deficiency with the higher elevations of methionine are due in part to lack of AdoMet. Such a suggestion, if correct, would provide not only an explanation of the CNS pathology, but also indicate why supplementation with AdoMet might be helpful.

### MAT I/III activity

#### Is there a relationship between the severity of loss of MAT I/III activity and the presence of CNS problems?

No such relationship is clearly established. Determination of the *MAT1A* genotype for a given patient does not provide a firm basis on which to judge the likelihood of CNS damage. A patient homozygous for truncating mutations that presumably leave no residual MAT I/III activity do not have CNS problems (e.g., Patient #2). Several compound heterozygotes with mutations that, when expressed individually in *E. coli*, have at least some residual activities [25–27, 34] do have CNS problems (e.g., patients 5, 9, 13, 20, 30, 31, 36, 37, 38, 39, 46, 60). One possibility is that more severe loss of MAT I/III activity and the resulting decrease in the rate of synthesis of AdoMet might lead to lowering the level of plasma AdoMet, thereby decreasing delivery of AdoMet from plasma to brain. However Fig. 3 shows that there is no trend for those with higher plasma methionines to have lower plasma AdoMet concentrations. Presumably plasma AdoMet is sustained at normal (93 ± 16 nM) or close to normal by MAT II activity even when MAT I/III activity is low or negligible (note that MAT II expression and protein are increased three-fold in *MAT1A* KO mice [54]). Figure 3 provides strong evidence that the CNS damage is not the result of differential delivery from plasma to brain at the concentrations that occur without AdoMet supplementation. It does not rule out the possibility that at higher concentrations of AdoMet resulting from supplementation, there might still be a therapeutic increase in the delivery of AdoMet to brain. Orally administered AdoMet has been shown to raise the plasma [73, 74] and the CSF [74] concentration of AdoMet. Because in most cases AdoMet supplementation has had positive therapeutic effects (Table 3), this method of therapy is supported by the available evidence.

### Does loss of hepatic MAT I/III activity impair the synthesis, and subsequent delivery from liver to brain of one or more compound(s) important in CNS function?

This possibility was considered previously for creatine and choline, formed in liver by the AdoMet-dependent guanidinoacetate and phosphatidylethanolamine methyltransferases [75], and appeared to be supported when early genetic studies found that two patients with brain demyelination were homozygotes for truncating *MAT1A* mutations that led to complete loss of MAT I/III activity [26]. However, the subsequent finding of a homozygous *MAT1A* mutation in a clinically well older patient (Patient #2) weakened the hypothesis in question and the data in Table 1 showing a lack of correlation between complete loss of activity and CNS problems further weaken it.

Another possibility related to decreased flux through phosphatidylethanolamine methyltransferase is that such decreases lead to CNS damage that may be prevented by docosahexaenoic acid (DHA) [76], a compound important in normal brain development and one the dietary intake of which is highly variable [77]. The intakes of DHA, and/or sources of choline, and creatine at crucial periods during growth, may therefore have played roles in the presence or absence of CNS problems in the MAT I/III patients. Variability in the CNS status of the three patients homozygous for R199C (patients 10, 11, and 13) might possibly be an example of effects of such factors (see, also the discussion [78]).

#### Does MAT I/III activity in brain contribute so significantly to the CNS need for AdoMet that more severe losses lead to the CNS problems?

Reytor et al. have shown that *MAT1A* is expressed in brain [12] but the expression in brain is very low (perhaps 0.2 %) of that in liver. It remains unclear what the total requirement of brain for AdoMet is, or the extent to which that requirement is met by MAT II. There is also the possibility that expressed *MAT1A* product affects nuclear localization or plays a role in, for example, specialized histone methylations [12], so a definitive answer to the question of the role of *MAT1A* expression in brain is not available.

### Do the increased levels of tHcy lead to the CNS problem?

As shown in Fig. 4, and consistent with previous observations [49], tHcy tends to rise as methionine increases. However, there is no striking elevation of the tHcy values for those with CNS problems above the tHcy values for those without CNS problems. Furthermore the tHcy levels are not higher than the values found in treated cystathionine β-synthase (CBS) deficient patients who have essentially normal IQ’s [79].

## Summary conclusions and tentative suggestions for management

No definitive answer to the question of what is the best strategy for management of severe MAT I/III deficiency emerges from consideration of either the available evidence on treatment outcomes or the numerous pathophysiologic mechanisms through which either loss of MAT I/III activity, methionine elevation, or some combination of both might lead to CNS problems. Given these givens, we suggest here what seem at this time to be reasonable, although tentative, management guidelines:

In an asymptomatic, well looking newborn of normal birthweight and gestational age with persistent hypermethioninemia and plasma tHcy higher than, perhaps 100 μM, CBS deficiency becomes the presumptive diagnosis. If, at most, plasma tHcy is elevated to perhaps 50–60 μM, the diagnosis is less clear. Determining the concentrations of AdoMet and AdoHcy in plasma of the patient, and, if possible, methionine in both parents will further pin-point the defect. To rule out CBS deficiency one may also determine cystathionine but this requires a suitably sensitive assay available in only a few specialized laboratories” [51]. Determination of the CBS activity in cultured fibroblasts and sequencing of the CBS gene will provide a final diagnosis. In the differential diagnosis of hypermethioninemia also deficiencies of S-adenosylhomocysteine hydrolase, adenosine kinase and glycine N-methyltransferase need to be mentioned. These inborn errors of methionine metabolism may also show elevated tHcy. The determination of AdoMet and AdoHcy is most helpful in pinpointing the potential defect. Secondary causes of hypermethioninemia include liver disease, tyrosinemia type I, galactosemia and citrin deficiency.

The majority of newborns now being flagged for methionine elevations in screening programs with lowered methionine cut-off values are turning out to be heterozygotes for one of the *MAT1A* mutations now known to cause mild to moderate hypermethioninemia. This heterozygosity will almost surely cause no adverse long-range clinical effects. If neither parent has a mild methionine elevation, and a sample up to three months has no more than a slightly high concentration of AdoMet at a time the methionine remains high, a diagnosis of homozygous or compound heterozygous MAT I/III deficiency is strongly indicated. Sequencing of the *MAT1A* gene will help to make the diagnosis more specific and secure. While these steps are being taken, the newborn might be monitored by assay of plasma methionine at about 1 month intervals. If that mean is above perhaps 500–600 μM, dietary methionine restriction should be considered, strict enough to maintain a methionine concentration near that level, but not a great deal below it. In patients with Mudd’s disease methionine levels may vary wide in the first months of life making the decision on methionine restriction not straightforward in this period. If possible this decision can better be made after the first months of life. If the mean plasma methionine is below 500 μM it seems likely that dietary restriction is not indicated.

Whether on methionine restriction or not, an eye should be kept out for development of CNS abnormalities and, if indicated, brain MRI performed. If problems arise while on a normal diet, starting or restarting methionine restriction or AdoMet supplementation may be considered. If problems develop while on restriction, supplementation with AdoMet should be considered. The available evidence suggests that AdoMet supplementation may lead to normalization of even MRI changes that have already occurred.

It is important to emphasize, again, that the stratification into those with or without evidence of CNS abnormalities, upon which the above suggestions rely to provide guidance, may be affected by ascertainment bias, and that the management guidelines put forth here are tentative and will need to be reevaluated and perhaps modified as experience with MAT I/III deficiency is extended.

## Abbreviations

MAT: Methionine adenosyltransferase
CNS: Central nervous system
AdoMet: S-adenosylmethionine
AdoHcy: S-adenosylhomocysteine
CBS: Cystathionine beta-synthase
LNAA: Large neutral amino acid system
tHcy: Total homocysteine in plasma
NBS: Newborn screening
